# Supplementary material for: Exploring hydrophilic 2,2-di(indol-3-yl)ethanamine derivatives against Leishmania infantum
Source: PLoS One. 2024 Jun 13;19(6):e0301901. doi: 10.1371/journal.pone.0301901 (PMC11175440; doi:10.1371/journal.pone.0301901)
Supplement: S1 File — (PDF) [file pone.0301901.s001.pdf]

Exploring hydrophilic 2,2-di(indol-3-yl)ethanamine derivatives against *Leishmania infantum*

Alessia Centanni[1&], Aurora Diotallevi[2&], Gloria Buffi[2], Diego Olivieri[2], Nuno Santarém[3,4], Antti Lehtinen[1], Jari Yli-Kauhaluoma[1], Anabela Cordeiro-da-Silva[3,4], Paula Kiuru[1]*, Simone Lucarini[2]*, Luca Galluzzi[2]

[1] Drug Research Program, Division of Pharmaceutical Chemistry and Technology, Faculty of Pharmacy, FI-00014 University of Helsinki, Finland

[2] Department of Biomolecular Sciences, University of Urbino Carlo Bo, 61029 Urbino (PU), Italy

[3] Instituto de Investigação e Inovação em Saúde da Universidade do Porto, R. Alfredo Allen 208 4200-135, Porto, Portugal

[4] Departamento de Ciências Biológicas, Faculdade de Farmácia da Universidade do Porto (FFUP), 4150-180 Porto, Portugal

* Corresponding authors

e-mail: paula.kiuru@helsinki.fi (PK), simone.lucarini@uniurb.it (SL)

[&] These authors contributed equally to this work.

SUPPORTING INFORMATION

Contents:

1. Formulation studies S2
2. List of aldehydes, carboxylic acids and aryl sulfonyl chlorides utilized for the synthesis of compounds **2-4** S4
3. Toxicity evaluation of compounds on THP-1 cells S5
4. Experimental data for URB1483 and compounds **1-4** S9
5. References S24

1. Formulation studies

Equivalence calculation between URB1483 concentration and injectable dose (mg/kg)

Considering an average mice weight of 25 g and an IP injectable volume of 100 µL, the following correspondence can be considered:

Dose (mg/kg)	[URB1483] (mg/mL)	[URB1483] (mM)
1	0.25	0.50
2	0.50	1.00
5	1.25	2.51
10	2.50	5.02

$$[URB1483](mg/mL) = \frac{Dose\ (mg/kg) \cdot 0.025\ (kg)}{0.1\ (mL)}$$

$$[URB1483](mM) = \frac{Dose\ (mg/kg) \cdot 0.025\ (kg)}{0.1\ (mL) \cdot 497.59(g/mol)}$$

MW URB1483 = 497.59 g·mol^{-1}

General procedure for the formulation study [1-3]

To the indicated volume of the URB1483 stock solution in DMSO (25.0 mg/mL, 50.24 mM) were added the appropriate volume or weight of excipient and the resulting mixture was heated at 37 °C and sonicated for 10-20 min. Finally, distilled water was added to reach the final volume of 1 mL (Table S1). The resulting formulation was maintained at 37 °C under stirring for 48-72 h. At regular time points, aliquots of the described formulations were sampled, centrifuged (8000 rpm, 10 min), and two volumes of acetonitrile were added. After further centrifugation (8000 rpm, 10 min), the resulted clear samples were analyzed by HPLC-ESI-MS for a percentage of the remaining compound. URB1483 was considered soluble and stable in the formulation with more than 90% of the compound remaining unaltered.

Table S1 – Preliminary Formulation Study on URB1483[a]

Entry	Final Dose	Stock Solution URB1483 Volume	Excipient	Solubility[b]
1[c]	1 mg/kg	5 µL	-	×
2[c]	1 mg/kg	5 µL	DMSO (95 µL)	×
3[c]	1 mg/kg	5 µL	DMSO (195 µL)	✓
4[c]	2 mg/kg	10 µL	DMSO (190 µL)	×
5	1 mg/kg	5 µL	PG (300 mg)	×
6	1 mg/kg	5 µL	PG (600 mg)	✓
7	2 mg/kg	10 µL	PG (600 mg)	×
8	1 mg/kg	5 µL	PEG 400 (300 mg)	×
9	1 mg/kg	5 µL	PEG 400 (400 mg)	✓
10	2 mg/kg	10 µL	PEG 400 (400 mg)	×
11	1 mg/kg	5 µL	PEG 400 (500 mg)	✓
12	2 mg/kg	10 µL	PEG 400 (500 mg)	✓
13	5 mg/kg	25 µL	PEG 400 (500 mg)	×
14	1 mg/kg	5 µL	PEG 400 (600 mg)	✓
15	2 mg/kg	10 µL	PEG 400 (600 mg)	✓
**16**	**5 mg/kg**	**25 µL**	**PEG 400 (600 mg)**	**✓**
17	10 mg/kg	50 µL	PEG 400 (600 mg)	×
18	1 mg/kg	5 µL	HPBC (200 mg)	×
19	1 mg/kg	5 µL	HPBC (400 mg)	✓
20	2 mg/kg	10 µL	HPBC (400 mg)	×

[a] DMSO = dimethylsulfoxide; PG = propylene glycol; PEG = polyethylene glycol; HPBC = 2-hydropropyl-β-cyclodextrin. [b] URB1483 was considered soluble and stable with more than 90% of the compound remaining unaltered after 48-72 h at 37 °C, by HPLC-ESI-MS analysis. [c] Instead of water, a physiological solution (saline) was utilized.

2. List of aldehydes, carboxylic acids and aryl sulfonyl chlorides utilized for the synthesis of compounds 2-4

For the synthesis of compounds **2**, the following aldehydes have been utilized

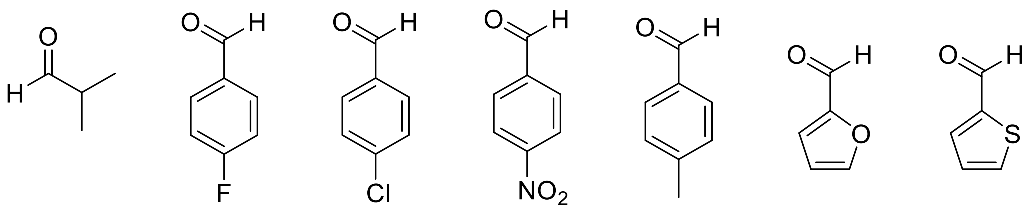

For the synthesis of compounds **3**, the following carboxylic acids have been utilized

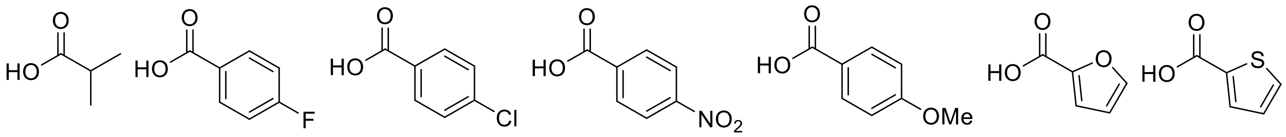

For the synthesis of compounds **4**, the following aryl sulfonyl chlorides have been utilized

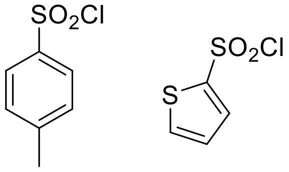

3. Toxicity evaluation of compounds on THP-1 cells

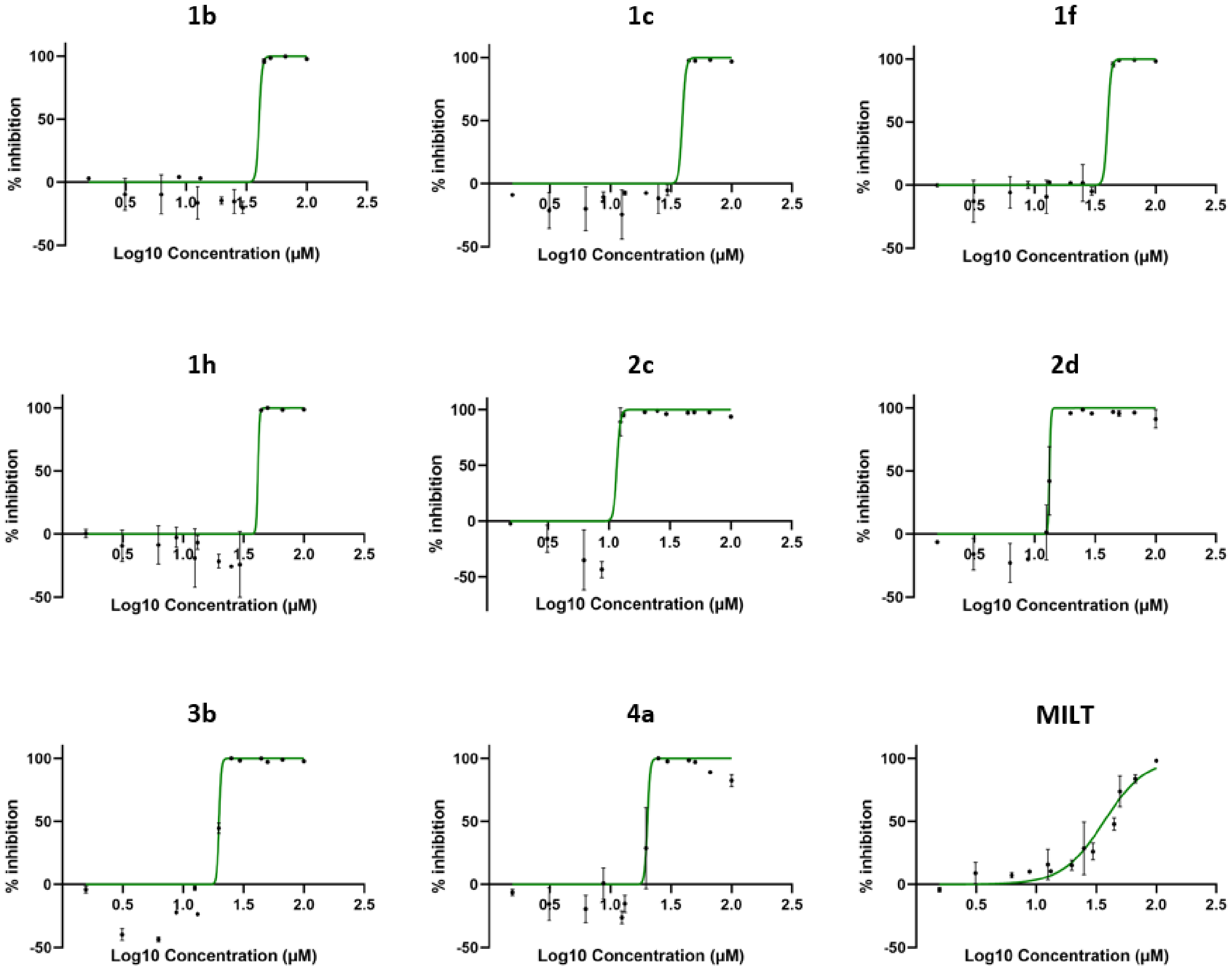

Figure S1 –Dose response curves (from 1.56 μM to 100 μM) of compounds **1b**, **1c**, **1f**, **1h**, **2c**, **2d**, **3b**, **4a** and miltefosine (MILT) on THP-1 cells.

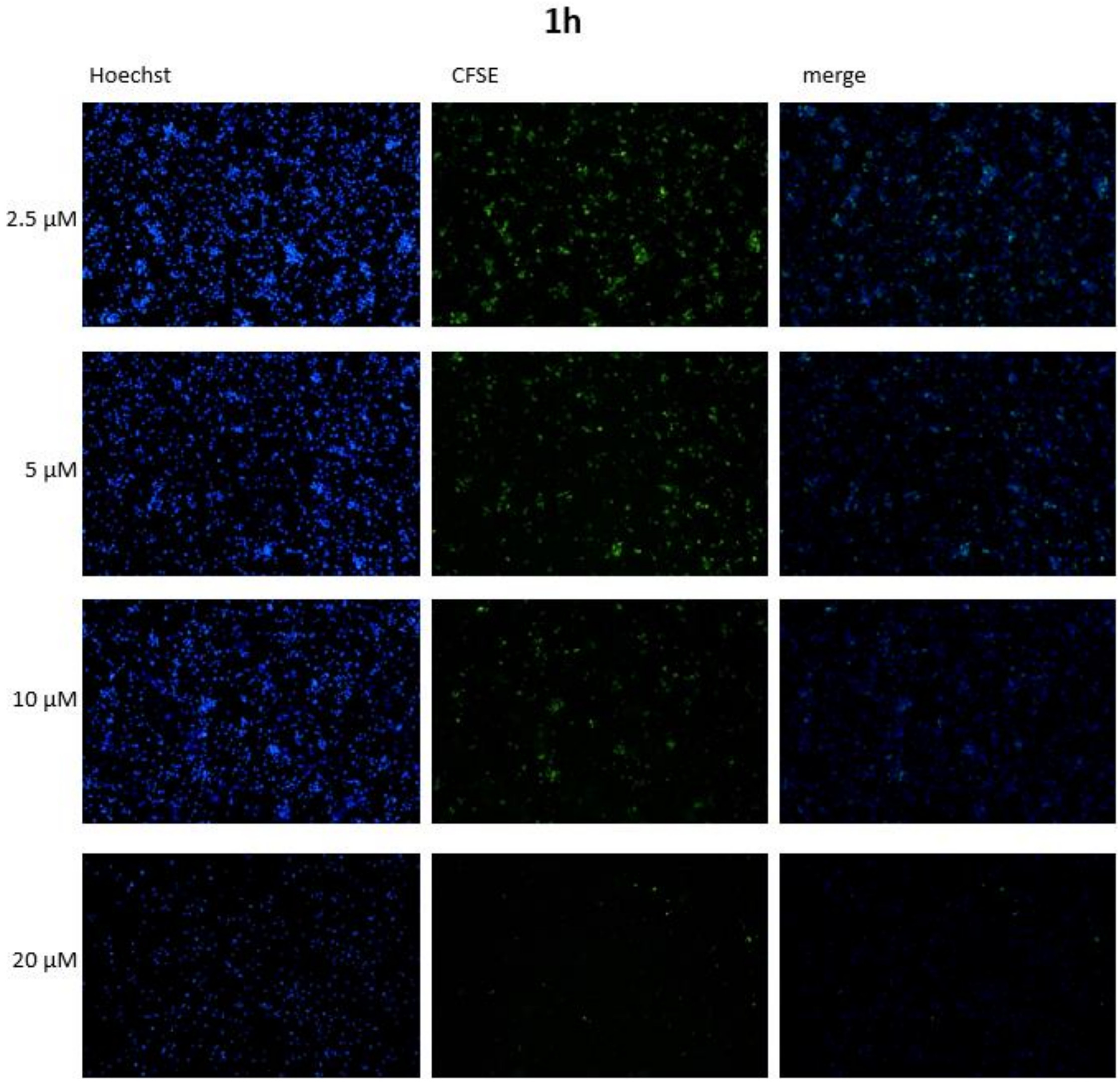

Figure S2 – Representative images (5× magnification) of THP-1 cells infected with *L. infantum* MHOM/TN/80/IPT1 for 24 h at 37°C and treated with compound **1h** (2.5 µM to 20 µM) for 72 h. *L. infantum* promastigotes were stained with carboxyfluorescein succinimidyl ester (CFSE) before infection; cell nuclei were stained with Hoechst dye.

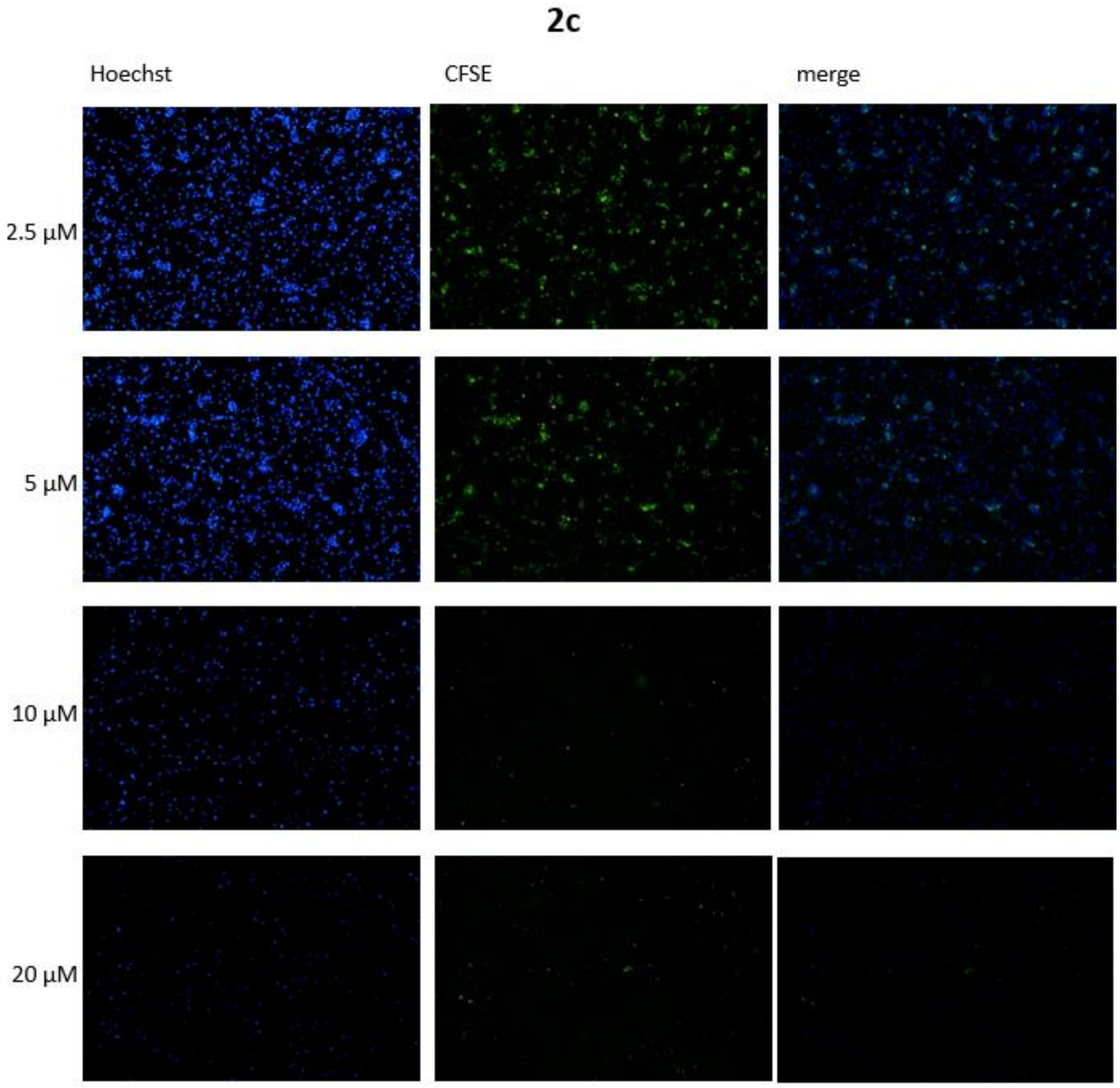

Figure S3 –Representative images (5× magnification) of THP-1 cells infected with *L. infantum* MHOM/TN/80/IPT1 for 24 h at 37°C and treated with compound **2c** (2.5 µM to 20 µM) for 72 h. *L. infantum* promastigotes were stained with carboxyfluorescein succinimidyl ester (CFSE) before infection; cell nuclei were stained with Hoechst dye.

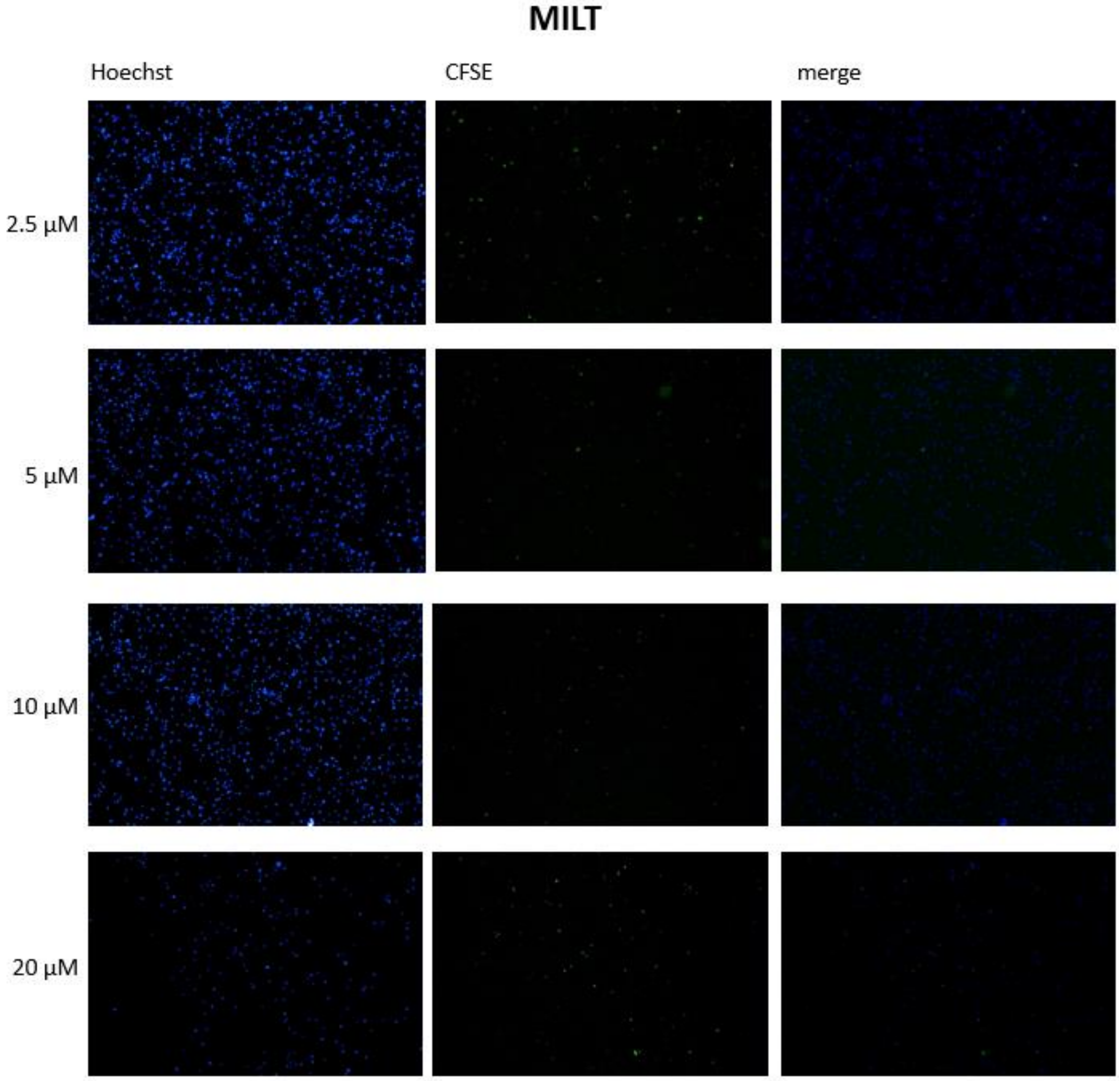

Figure S4 –Representative images (5× magnification) of THP-1 cells infected with *L. infantum* MHOM/TN/80/IPT1 for 24 h at 37°C and treated with MILT (2.5 µM to 20 µM) for 72 h. *L. infantum* promastigotes were stained with carboxyfluorescein succinimidyl ester (CFSE) before infection; cell nuclei were stained with Hoechst dye.

4. Experimental data for URB1483 and compounds 1-4

All the compounds were synthesized following the general procedures **A**, **B**, **C** or **D**, reported in the main article (materials and methods, chemistry section).

3-Ethyl 4-methyl 1-[2,2-bis(1-methyl-1*H*-indol-3-yl)ethyl]-2-methyl-1*H*-pyrrole-3,4-dicarboxylate (URB1483)

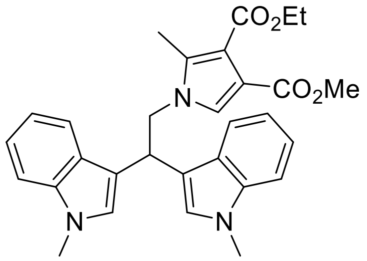

URB1483 was synthesized following a reported literature procedure [4], and it was isolated by column chromatography as a pale tallow solid (EtOAc/cyclohexane 35:65) in 39% yield (102 mg). ^{1}H NMR (400 MHz, DMSO) δ 7.55 (d, *J* = 8.0 Hz, 2H), 7.35 (d, *J* = 8.0 Hz, 2H), 7.33 (s, 2H), 7.22 (s, 1H), 7.09 (t, *J* = 7.6 Hz, 2H), 6.94 (t, *J* = 7.6 Hz, 2H), 4.91 (t, *J* = 7.6 Hz, 1H), 4.61 (d, *J* = 7.6 Hz, 2H), 4.10 (q, *J* = 7.2 Hz, 2H), 3.72 (s, 6H), 3.60 (s, 3H), 2.19 (s, 3H), 1.19 (t, *J* = 7.2 Hz, 3H). Anal. calcd. for $C_{30}H_{31}N_3O_4$ (497.58) C 72.41, H 6.28, N 8.44; found C 72.55, H 6.31, N 8.55. Data for compound URB1483 are in agreement with those reported in the literature.[4]

2,2-Di(1*H*-indol-3-yl)ethan-1-amine (1a)

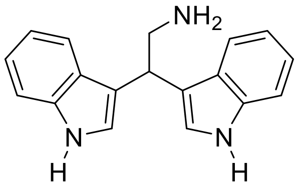

Following the general procedure **A**, compound **1a** was isolated by column chromatography as a white solid (DCM/MeOH/TEA, 90:9:1) in 55% overall yield (61 mg). ^{1}H NMR (400 MHz, DMSO) δ 10.71 (br. s, 2H), 7.47 (d, *J* = 8.0 Hz, 2H), 7.28 (d, *J* = 8.0 Hz, 2H), 7.14 (d, *J* = 2.0 Hz, 2H), 6.98 (app t, *J* = 8.0 Hz, 2H), 6.85 (app t, *J* = 8.0 Hz, 2H), 4.69 (app t, *J* = 7.0 Hz, 1H), 3.88 (d, *J* = 7.0 Hz, 2H). Anal. calcd. for $C_{18}H_{17}N_3$ (275.35) C 78.52, H 6.22. N 15.26; found C 78.67, H 6.16. N 15.33. Data for compound **1a** are in agreement with those reported in the literature. [5]

2,2-bis(6-Fluoro-1*H*-indol-3-yl)ethan-1-amine (1b)

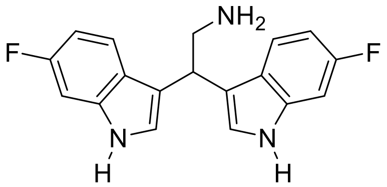

Following the general procedure **A**, compound **1b** was isolated by column chromatography as white solid (DCM/MeOH/TEA, 90:9:1) in 70% overall yield (87 mg). ^{1}H NMR (400 MHz, CD_3OD) δ 7.44 (dd, J = 9.0, 5.0 Hz, 2H), 7.14 (d, J = 3.0 Hz, 2H), 7.04 (dd, J = 9.5, 2.0 Hz, 2H), 6.72 (ddd, J = 9.5, 9.0, 2.0 Hz, 2H), 4.52 (app t, J = 7.5 Hz, 1H), 3.37-3.41 (m, 2H). Anal. calcd. for $C_{18}H_{15}F_2N_3$ (311.33) C 69.44, H 4.86. N 13.50; found C 69.58, H 4.91. N 13.60. Data for compound **1b** are in agreement with those reported in the literature. [6]

2,2-bis(6-Chloro-1*H*-indol-3-yl)ethan-1-amine (1c)

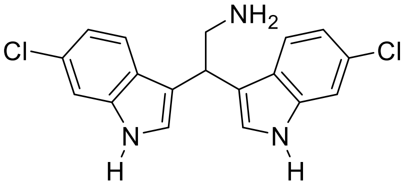

Following the general procedure **A**, compound **1c** was isolated by column chromatography as brownish solid (DCM/MeOH 100:0 to 80:20) in 42% overall yield (58 mg). ^{1}H NMR (400 MHz, $CDCl_3$) δ 8.20 (s, 2H), 7.42 (d, J = 8.5 Hz, 2H), 7.33 (d, J = 1.8 Hz, 2H), 7.02 (d, J = 2.3 Hz, 2H), 6.99 (dd, J = 8.5, 1.8 Hz, 2H), 4.47 (t, J = 7.0 Hz, 1H), 3.42 (d, J = 7.0 Hz, 2H). ^{13}C NMR (101 MHz, $CDCl_3$) δ 137,1, 128.1, 125.7, 122.6, 120.4, 120.2, 117.7, 111.3, 46.5, 38.1. HRMS (TOF-ESI$^+$): m/z calcd for $C_{18}H_{16}N_3Cl_2$ $[M + H]^+$ 344.0721; found 344.0722. LC-MS: $[M -$ 6-chloroindole$]^+$ m/z 193 (t_r = 3.29 min), >99%.

2,2-bis(6-Bromo-1*H*-indol-3-yl)ethan-1-amine (1d)

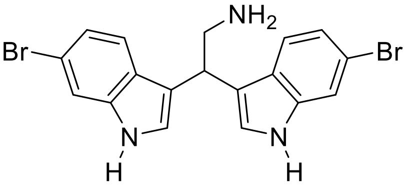

Following the general procedure **A**, compound **1d** was isolated by column chromatography as white solid (DCM/MeOH 100:0 to 80:20) in 71% overall yield (1.39 g). ^{1}H NMR (400 MHz, CD_3OD) δ 7.50 (dd, J = 1.8, 0.5 Hz, 2H), 7.38 (dd, J = 8.5, 0.5 Hz, 2H), 7.14 (d, J = 0.8 Hz, 2H), 7.03 (dd, J = 8.5, 1.8 Hz, 2H), 4.53 (app t, J = 7.5 Hz, 1H), 3.39 (d, J = 7.5 Hz, 2H). LC-MS: HRMS (TOF-ESI$^+$):

m/z calcd for $C_{18}H_{16}N_3Br_2$ $[M+H]^+$ 431.9711; found 431.9710. [M - 6-bromoindole]$^+$ m/z 272 (t_r = 3.60 min), >99%. Data for compound **1d** are in agreement with those reported in the literature. [5**]**

2,2-bis(5-Iodo-1*H*-indol-3-yl)ethan-1-amine (1e)

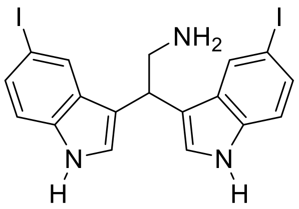

Following the general procedure **A**, compound **1e** was isolated by column chromatography as brownish solid (DCM/MeOH 100:0 to 80:20) in 57% overall yield (120 mg). ^{1}H NMR (400 MHz, $CDCl_3$) δ 8.12 (br s, 2H), 7.90 (d, J = 1.6 Hz, 2H), 7.42 (dd, J = 8.5, 1.6 Hz, 2H), 7.15 (d, J = 8.5 Hz, 2H), 7.05-7.02 (m, 2H), 4.42 (t, J = 7.0 Hz, 1H), 3.42 (d, J = 7.0 Hz, 2H). ^{13}C NMR (101 MHz, $CDCl_3$) δ 135.9, 130.7, 129.6, 128.5, 122.9, 116.9, 113.4, 83.1, 46.5, 38.2. HRMS (TOF-ESI$^+$): m/z calcd for $C_{18}H_{16}N_3I_2$ $[M+H]^+$ 527.9434; found 527.9432. LC-MS: [M - 5-iodoindole]$^+$ m/z 285 (t_r = 3.74 min), >99%.

Dimethyl 3,3'-(2-aminoethane-1,1-diyl)bis(1*H*-indole-6-carboxylate) (1f)

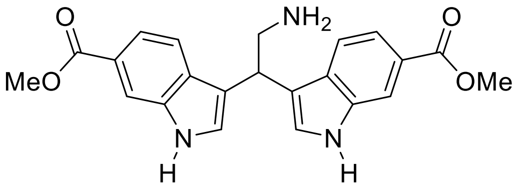

Following the general procedure **A**, compound **1f** was isolated by column chromatography as off-white solid (direct phase: DCM/MeOH 100:0 to 93:7, then reverse èhase: MeOH/H_2O 100:0 to 0:100) in 5% overall yield (10 mg). ^{1}H NMR (400 MHz, CD_3OD) δ 8.08 (dd, J = 1.5, 0.7 Hz, 2H), 7.61 (dd, J = 8.5, 1.5 Hz, 2H), 7.55 (dd, J = 8.5, 0.7 Hz, 2H), 7.37 (s, 2H), 4.62 (t, J = 7.4 Hz, 1H), 3.88 (s, 6H), 3.43 (s, 2H). ^{13}C NMR (101 MHz, CD_3OD) δ 170.0, 137.7, 131.9, 127.4, 124.0, 120.6, 119.6, 118.0, 114.8, 52.3, 46.9, 38.2. HRMS (TOF-ESI$^+$): m/z calcd for $C_{22}H_{22}N_3O_4$ $[M + H]^+$ 392.1610; found 392.1613. LC-MS: [M – indole-6-carboxylate]$^+$ m/z 217 (t_r = 2.45 min), >99%.

3,3'-(2-Aminoethane-1,1-diyl)bis(1*H*-indole-5-carbonitrile) (1g)

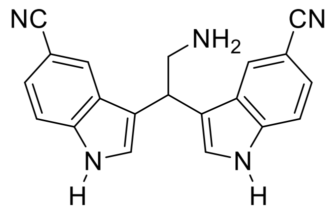

Following the general procedure **A**, compound **1h** was isolated by column chromatography as white solid (DCM/MeOH 100:0 to 50:50) in 56% overall yield (56 mg). ^{1}H NMR (400 MHz, CD_3OD) δ 7.85 (dd, J = 1.6, 0.7 Hz, 2H), 7.47 (dd, J = 8.5, 0.7 Hz, 2H), 7.40 (d, J = 0.8 Hz, 2H), 7.32 (dd, J = 8.5, 1.6 Hz, 2H), 4.61-4.55 (m, 1H), 3.40 (d, J = 7.4 Hz, 2H). ^{13}C NMR (101 MHz, CD_3OD) δ 140.3, 128.0, 126.1, 125.8, 125.2, 121.9, 118.4, 113.6, 102.3, 46.8, 38.4. HRMS (TOF-ESI^{+}): *m/z* calcd for $C_{20}H_{16}N_5$ [M + H]$^{+}$ 326.1404; found 326.1404. LC-MS: [M - 5-cyanoindole]$^{+}$ *m/z* 184 (t_r = 2.15 min), >99%.

2,2-bis(1-Methyl-1*H*-indol-3-yl)ethan-1-amine (1h)

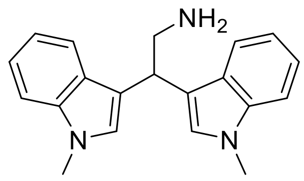

Following the general procedure **A**, compound **1h** was isolated by column chromatography as white solid (DCM/MeOH/TEA, 90:9:1) in 51% overall yield (62 mg). ^{1}H NMR (400 MHz, CD_3OD) δ 7.51 (d, J = 7.5 Hz, 2H), 7.16 (d, J = 7.5 Hz, 2H), 7.07 (app t, J = 7.5 Hz, 2H), 6.92 (dd, J = 7.5 Hz, 2H), 6.86 (s, 2H), 4.48 (app t, J = 7.5 Hz, 1H), 3.46 (s, 6 H), 3.29 (d, J = 7.5 Hz, 2H). Anal. calcd. for $C_{20}H_{21}N_3$(303.40) C 79.17, H 6.98, N 13.85; found C 79.07, H 7.04, N 13.91. Data for compound **1h** are in agreement with those reported in the literature. [4]

2,2-bis(6-Bromo-1-methyl-1*H*-indol-3-yl)ethan-1-amine (1i)

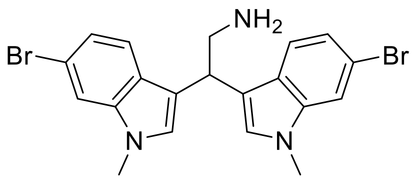

Following the general procedure **A**, compound **1i** was isolated by column chromatography as yellowish solid (DCM/MeOH/TEA, 90:9:1) in 77% overall yield (142 mg). ^{1}H NMR (400 MHz, CD_3OD) δ 7.51 (d, J = 1.5 Hz, 2H), 7.40 (d, J = 8.5 Hz, 2H), 7.06 (dd, J = 8.5, 1.5 Hz, 2H), 7.06 (s, 2H), 4.49 (app t, J = 7.5 Hz, 1H), 3.72 (s, 6H), 3.32 (d, J = 7.5 Hz, 2H). Anal. calcd. for

$C_{20}H_{19}Br_2N_3$(461.19): C 52.09, H 4.15, N 9.11; found: C 52.21, H 4.08, N 9.19. Data for compound **1i** are in agreement with those reported in the literature. [4]

N-[2,2-bis(6-Bromo-1H-indol-3-yl)ethyl]-2-methylpropan-1-amine (2a)

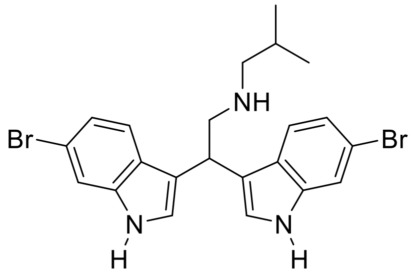

Following the general procedure **B**, compound **2a** was isolated as a white solid (DCM/MeOH 100:0 to 80:20) in 82% yield (80 mg). ^{1}H NMR (400 MHz, CD_3OD) δ 7.51 (dd, J = 1.8, 0.3 Hz, 2H), 7.40 (dd, J = 8.5, 0.6 Hz, 2H), 7.14 (d, J = 0.8 Hz, 2H), 7.03 (dd, J = 8.5, 1.8 Hz, 2H), 4.68 (app t, J = 7.6 Hz, 1H), 3.31 (d, J = 7.6 Hz 2H), 2.46 (d, J = 6.9 Hz, 2H), 1.70-1.62 (m, 1H), 0.75 (d, J = 6.6 Hz, 6H). ^{13}C NMR (101 MHz, CD_3OD) δ 139.2, 127.0, 124.1, 122.7, 121.3, 117.8, 115.9, 115.2, 58.3, 54.8, 34.9, 28.6, 20.7. HRMS (TOF-ESI$^+$): *m/z* calcd for $C_{22}H_{24}N_3Br_2$ $[M+H]^+$ 488.0337; found 488.0338. LC-MS: $[M + 2 + H]^+$ *m/z* 490 (t_r = 4.26 min), >99%.

2,2-bis(6-Bromo-1H-indol-3-yl)-N-(4-fluorobenzyl)ethan-1-amine (2b)

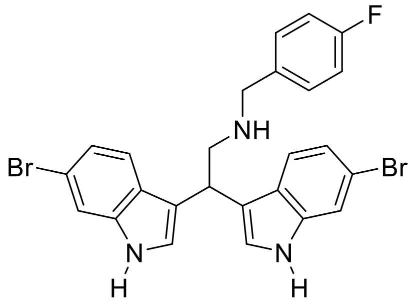

Following the general procedure **B**, compound **2b** was isolated as a white solid (DCM/MeOH 100:0 to 80:20) in 40% yield (45 mg). ^{1}H NMR (400 MHz, $CDCl_3$) δ 8.03 (br s, 2H), 7.48 (d, J = 1.6 Hz, 2H), 7.36 (d, J = 8.5 Hz, 2H), 7.21-7.15 (m, 2H), 7.12 (dd, J = 8.5, 1.7 Hz, 2H), 6.99-6.91 (m, 4H), 4.67 (app t, J = 7.1 Hz, 1H), 3.79 (s, 2H), 3.33 (d, J = 7.0 Hz, 2H), ^{13}C NMR (101 MHz, $CDCl_3$) δ 162.0 (d, $J_{C,F}$ = 245 Hz), 137.5, 136.04, 136.01, 129.7 (d, $J_{C,F}$ = 8 Hz), 125.9, 122.7 (d, $J_{C,F}$ = 23 Hz), 120.8, 118.0, 115.9, 115.3 (d, $J_{C,F}$ = 21 Hz), 114.3, 53.2 (2C), 34.5. HRMS (TOF-ESI$^+$): *m/z* calcd for $C_{25}H_{21}N_3Br_2F$ $[M + H]^+$ 540.0086; found: 540.0085. LC-MS: $[M + 2+ H]^+$ *m/z* 542 (t_r = 4.41 min), >96%.

2,2-bis(6-Bromo-1*H*-indol-3-yl)-*N*-(4-chlorobenzyl)ethan-1-amine (2c)

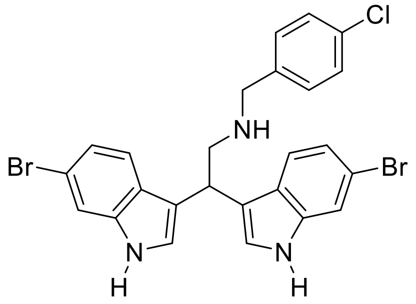

Following the general procedure **B**, compound **2c** was isolated as an off-white solid (DCM/MeOH 100:0 to 80:20) in 48% yield (80 mg). ^{1}H NMR (400 MHz, $CDCl_3$) δ 8.02 (br s, 2H), 7.44 (d, J = 1.7 Hz, 2H), 7.35 (d, J = 8.5 Hz, 2H), 7.25 – 7.20 (m, 2H), 7.17 – 7.09 (m, 4H), 6.98 (dd, J = 2.4, 0.9 Hz, 2H), 4.68 (app t, J = 7.0 Hz, 1H), 3.79 (s, 2H), 3.32 (d, J = 7.0 Hz, 2H). ^{13}C NMR (101 MHz, $CDCl_3$) δ 138.7, 137.5, 132.8, 129.5, 128.6, 125.8, 122.9, 122.6, 120.8, 117.9, 115.9, 114.3, 53.1 (2C), 34.5. HRMS (TOF-ESI$^+$): *m/z* calcd for $C_{25}H_{21}N_3ClBr_2$ [M + H]$^+$ 555.9791; found 555.9793. LC-MS: [M + 2 + H]$^+$ *m/z* 558 (t_r = 4.64 min), >99%.

2,2-bis(6-Bromo-1*H*-indol-3-yl)-*N*-(4-nitrobenzyl)ethan-1-amine (2d)

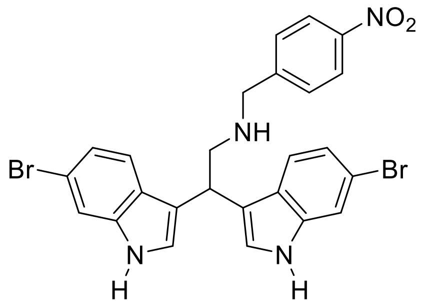

Following the general procedure **B**, compound **2d** was isolated as a pale yellow solid (*n*-heptane/EtOAc 100:0 to 25:75) in 58% yield (66 mg). ^{1}H NMR (400 MHz, $CDCl_3$) δ 8.17-8.07 (m, 2H), 8.03 (br s, 2H), 7.50 (d, J = 1.7 Hz, 2H), 7.42-7.33 (m, 4H), 7.12 (dd, J = 8.5, 1.7 Hz, 2H), 7.02 (dd, J = 2.4, 0.8 Hz, 2H), 4.71 (t, J = 6.9 Hz, 1H), 3.91 (s, 2H), 3.34 (d, J = 6.9 Hz, 2H). ^{13}C NMR (101 MHz, $CDCl_3$) δ 148.3, 147.1, 137.5, 128.7, 125.8, 123.7, 122.9, 122.6, 120.7, 117.8, 116.0, 114.3, 53.5, 53.2, 34.6. HRMS (TOF-ESI$^+$): *m/z* calcd for $C_{25}H_{21}N_4Br_2O_2$ [M + H]$^+$ 567.0031; found 567.0033. LC-MS: [M + 2 + H]$^+$ *m/z* 569 (t_r = 4.33 min), >99%.

2,2-bis(6-Bromo-1*H*-indol-3-yl)-*N*-(4-methylbenzyl)ethan-1-amine (2e)

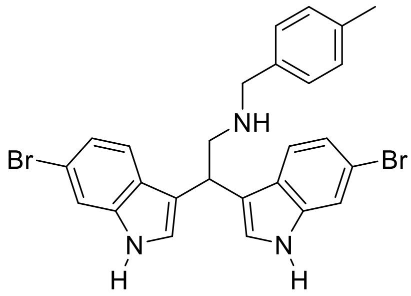

Following the general procedure **B**, compound **2e** was isolated as a white solid (DCM/MeOH 100:0 to 80:20) in 41% yield (66 mg). ^{1}H NMR (400 MHz, $CDCl_3$) δ 8.23 (br s, 2H), 7.40 (d, J = 1.6 Hz, 2H), 7.32 (d, J = 8.5 Hz, 2H), 7.16-7.02 (m, 6H), 6.93-6.74 (m, 2H), 4.69 (app t, J = 7.1 Hz, 1H), 3.80 (s, 2H), 3.35 (d, J = 7.1 Hz, 2H), 2.33 (s, 3H). ^{13}C NMR (101 MHz, $CDCl_3$) δ 137.4, 136.8, 129.2, 128.2, 125.8, 122.7 (2C), 120.7, 117.7, 115.7, 114.3, 53.5, 53.1, 34.4, 21.2. HRMS (TOF-ESI^{+}): m/z calcd for $C_{26}H_{24}N_3Br_2$ $[M + H]^+$ 536.0337; found 536.0338. LC-MS: $[M + 2 + H]^+$ m/z 538 (t_r = 4.62 min), >99%.

2,2-bis(6-Bromo-1*H*-indol-3-yl)-*N*-(furan-2-ylmethyl)ethan-1-amine (2f)

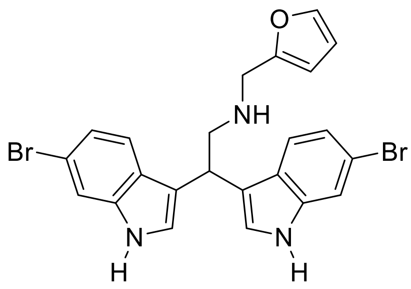

Following the general procedure **B**, compound **2f** was isolated as an off-white solid (DCM/MeOH 100:0 to 80:20) in 39% yield (59 mg). ^{1}H NMR (400 MHz, $CDCl_3$) δ 8.18 (br s, 2H), 7.44 (s, 2H), 7.35 (d, J = 8.5 Hz, 2H), 7.31 (d, J = 1.9 Hz, 1H), 7.11 (dd, J = 8.5, 1.7 Hz, 2H), 6.90 (s, 2H), 6.29 (dd, J = 3.2, 1.9 Hz, 1H), 6.12 (d, J = 3.2 Hz, 1H), 4.66 (t, J = 7.1 Hz, 1H), 3.82 (s, 2H), 3.35 (d, J = 7.1 Hz, 2H). ^{13}C NMR (101 MHz, $CDCl_3$) δ 153.8, 141.9, 137.5, 125.8, 122.8, 122.7, 120.8, 117.9, 115.8, 114.3, 110.3, 107.3, 53.0, 46.2, 34.5. HRMS (TOF-ESI^{+}): m/z calcd for $C_{23}H_{20}N_3OBr_2$ $[M + H]^+$ 511.9973; found 511.9976. LC-MS: $[M + 2 + H]^+$ m/z 514 (t_r = 4.11 min), >99%.

2,2-bis(6-Bromo-1*H*-indol-3-yl)-*N*-(thiophen-2-ylmethyl)ethan-1-amine (2g)

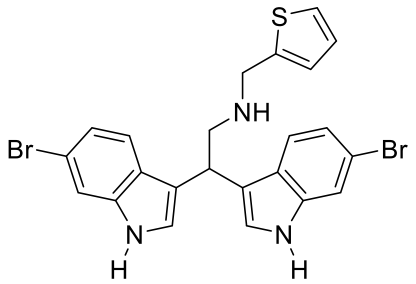

Following the general procedure **B**, compound **2g** was isolated as an off-white solid (DCM/MeOH 100:0 to 80:20) in 40% yield (60 mg). ^{1}H NMR (400 MHz, $CDCl_3$) δ 7.99 (br s, 2H), 7.49 (d, J = 1.7, 2H), 7.37 (d, J = 8.5, 2H), 7.19 (dd, J = 5.1, 1.2 Hz, 1H), 7.12 (dd, J = 8.5, 1.7 Hz, 2H), 7.01 (dd, J = 2.4, 0.9 Hz, 2H), 6.92 (dd, J = 5.1, 3.4 Hz, 1H), 6.86-6.83 (m, 1H), 4.71 (app t, J = 6.9 Hz, 1H), 4.02 (s, 2H), 3.39 (d, J = 6.9 Hz, 2H). ^{13}C NMR (101 MHz, $CDCl_3$) δ 144.3, 137.5, 126.7, 125.9, 125.0, 124.5, 122.8, 122.7, 120.8, 118.0, 115.8, 114.2, 53.1, 48.6, 34.4. HRMS (TOF-ESI$^+$): m/z calcd for $C_{23}H_{20}N_3SBr_2$ $[M + H]^+$ 527.9745; found 527.9746. LC-MS: $[M + 2 + H]^+$ m/z 530 (t_r = 4.25min), >99%.

N-[2,2-bis(6-Bromo-1H-indol-3-yl)ethyl]isobutyramide (3a)

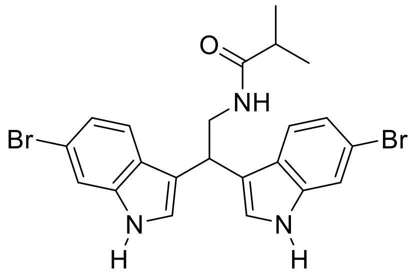

Following the general procedure **C**, compound **3a** was isolated as a white solid (DCM/MeOH 100:0 to 95:5) in 67% yield (65 mg). ^{1}H NMR (400 MHz, $(CD_3)_2CO$) δ 10.21 (br s, 2H), 7.57 (dd, J = 1.8, 0.5 Hz, 2H), 7.55-7.51 (m, 2H), 7.24-7.21 (m, 2H), 7.07 (dd, J = 8.5, 1.8 Hz, 2H), 4.75 (app t, J = 7.4 Hz, 1H), 3.93 (dd, J = 7.4, 6.0 Hz, 2H), 2.36-2.24 (m, 1H) 0.99 (d, J = 6.9 Hz, 6H). ^{13}C NMR (101 MHz, $(CD_3)_2CO$) δ 177.2, 138.8, 127.2, 124.4, 122.4, 121.7, 117.9, 115.2, 115.0, 44.6, 35.2, 34.6 19.5 (2C). HRMS (TOF-ESI$^+$): m/z calcd for $C_{14}H_{16}N_2OBr$ [M - 6-bromoindole]$^+$ 307.0446; found 307.0444. LC-MS: [M - 6-bromoindole]$^+$ m/z 307 (t_r = 5.19 min), >99%.

N-[2,2-bis(6-Bromo-1H-indol-3-yl)ethyl]-4-fluorobenzamide (3b)

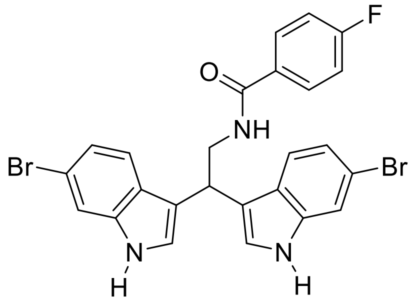

Following the general procedure **C**, compound **3b** was isolated as a white solid (n-hexane/acetone 90:10 to 44:66) in 45% yield (50 mg). ^{1}H NMR (400 MHz, $(CD_3)_2CO$) δ 10.23 (br s, 2H), 7.94-7.88 (m, 1H), 7.88-7.82 (m, 2H), 7.59-7.54 (m, 3H), 7.30 – 7.28 (m, 2H), 7.17 – 7.10 (m, 2H), 7.06 (dd, J = 8.5, 1.8 Hz, 2H), 4.94(t, J = 7.4 Hz, 1H), 4.15 (dd, J = 7.4, 5.9 Hz, 2H). ^{13}C NMR (101 MHz, $(CD_3)_2CO$) δ 166.6, 165.2 (d, $J_{C,F}$ = 249 Hz), 138.8, 132.5 (d, $J_{C,F}$ = 3 Hz), 130.6 (d, $J_{C,F}$ = 8 Hz), 127.2, 124.5, 122.4, 121.7, 117.9, 115.8 (d, $J_{C,F}$ = 22 Hz), 115.3, 115.1, 45.4, 35.1. HRMS (TOF-ESI$^+$): m/z calcd for $C_{17}H_{13}BrFN_2O$ [M – 6-bromoindole]$^+$ 359.0195; found 359.0195. LC-MS: [M + 2 + H]$^+$ m/z 359 (t_r = 5.81 min), >99%.

***N*-[2,2-bis(6-Bromo-1H-indol-3-yl)ethyl]-4-chlorobenzamide (3c)**

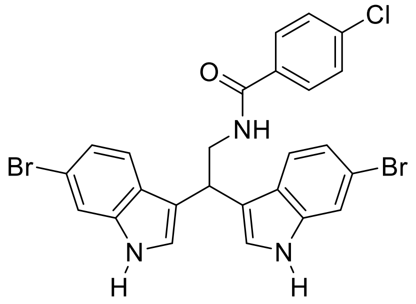

Following the general procedure **C**, compound **3c** was isolated as a white solid (DCM/MeOH 100:0 to 80:20) in 37% yield (43 mg). ^{1}H NMR (400 MHz, $(CD_3)_2CO$) δ 10.22 (br s, 2H), 8.06-7.89 (m, 1H), 7.8-7.73 (m, 2H), 7.61 – 7.51 (m, 4H), 7.44 – 7.37 (m, 2H), 7.33-7.24 (m, 2H), 7.06 (dd, J = 8.5, 1.8 Hz, 2H), 4.94 (app t, J = 7.4 Hz, 1H), 4.16 (dd, J = 7.4, 5.9 Hz, 2H). ^{13}C NMR (101 MHz, $(CD_3)_2CO$) δ 166.6, 138.8, 137.4, 134.8, 129.8, 129.2, 127.2, 124.5, 122.4, 121.7, 117.9, 115.3, 115.1, 45.4, 35.1. HRMS (TOF-ESI$^+$): *m/z* calcd for $C_{17}H_{13}BrClN_2O$ [M – 6-bromoindole]$^+$ 374.9900; found 374.9902. LC-MS: [M – 6-bromoindole +2]$^+$ *m/z* 377 (t_r = 5.97 min), >99%.

***N*-[2,2-bis(6-Bromo-1*H*-indol-3-yl)ethyl]-4-nitrobenzamide (3d)**

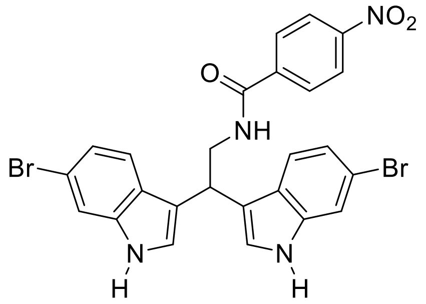

Following the general procedure **C**, compound **3d** was isolated as a yellow solid (*n*-heptane/EtOAc 100:0 to 25:75) in 45% yield (50 mg). ^{1}H NMR (400 MHz, $(CD_3)_2CO$) δ 10.23 (br s, 2H), 8.27-8.17 (m, 3H), 8.04-7.96 (m, 2H), 7.61 – 7.52 (m, 4H), 7.33-7.29 (m, 2H), 7.07 (dd, J = 8.5, 1.8 Hz, 2H), 4.97 (t, J = 7.5 Hz, 1H), 4.20 (dd, J = 7.5, 5.9 Hz, 2H). ^{13}C NMR (101 MHz, $(CD_3)_2CO$) δ 166.1, 150.3, 141.7, 138.8, 129.4, 127.1, 124.5, 124.2, 122.4, 121.7, 117.7, 115.3, 115.1, 45.6, 35.0. HRMS (TOF-ESI$^+$): *m/z* calcd for $C_{17}H_{13}BrN_3O_3$ [M + H]$^+$ 567.0031; found 567.0027. LC-MS: [M + 2]$^+$ *m/z* 569 (t_r = 4.32min), >99%.

N-[2,2-bis(6-Bromo-1H-indol-3-yl)ethyl]-4-methoxybenzamide (3e)

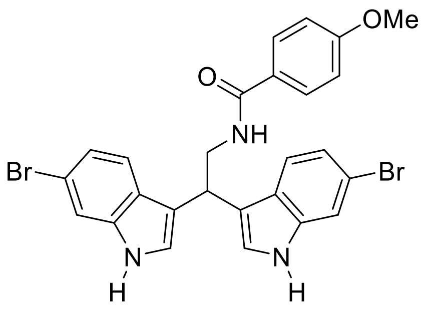

Following the general procedure **C**, compound **3e** was isolated as a white solid (DCM/MeOH 100:0 to 95:5) in 45% yield (50 mg). ^{1}H NMR (400 MHz, $(CD_3)_2CO$) δ 10.21 (br s, 2H), 7.76 (d, J = 8.9 Hz, 2H), 7.59 – 7.55 (m, 4H), 7.29 – 7.27 (m, 2H), 7.06 (dd, J = 8.6, 1.7 Hz, 2H), 6.90 (d, J = 8.9 Hz, 2H), 4.93 (app t, J = 7.4, 1H), 4.14 (dd, J = 7.4, 5.9 Hz, 2H), 3.81 (s, 3H). ^{13}C NMR (101 MHz, $(CD_3)_2CO$) δ 167.1, 162.9, 138.8, 129.7, 128.4, 127.2, 124.4, 122.4, 121.8, 118.0, 115.2, 115.0, 114.2, 55.7, 45.3, 35.2. HRMS (TOF-ESI$^+$): m/z calcd for $C_{18}H_{16}BrN_2O_2$ [M – 6-bromoindole]$^+$ 371.0395; found 371.0392. LC-MS: [M – 6-bromoindole]$^+$ m/z 371 (t_r = 5.51 min), >99%.

N-[2,2-bis(6-Bromo-1H-indol-3-yl)ethyl]furan-2-carboxamide (3f)

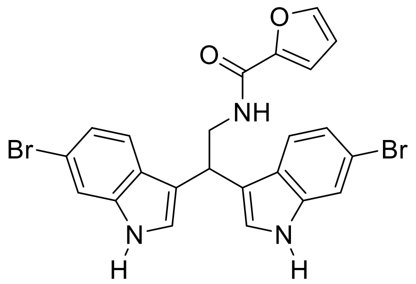

Following the general procedure **C**, compound **3f** was isolated as a white solid (DCM/MeOH 100:0 to 95:5) in 45% yield (45 mg). ^{1}H NMR (400 MHz, $(CD_3)_2CO$) δ 10.24 (br s, 2H), 7.71 – 7.61 (m, 1H), 7.60 – 7.53 (m, 5H), 7.32-7.27 (m, 2H), 7.07 (dd, J = 8.5, 1.8 Hz, 2H), 7.00 (dd, J = 3.4, 0.9 Hz, 1H), 6.51 (dd, J = 3.4, 1.8 Hz, 1H), 4.95 (app t, J = 7.4 Hz, , 1H), 4.14 (dd, J = 7.4, 6.0 Hz, 2H). ^{13}C NMR (101 MHz, $(CD_3)_2CO$) δ 159.0, 149.6, 145.1, 138.8, 127.1, 124.4, 122.4, 121.7, 117.8, 115.3, 115.1, 114.0, 112.6, 44.4, 23.3. HRMS (TOF-ESI$^+$): m/z calcd for $C_{15}H_{12}BrN_2O_2$ [M - 6-bromoindole]$^+$ 331.0082; found 331.0083. LC-MS: [M - 6-bromoindole]$^+$ m/z 331 (t_r = 5.23 min), >99%.

_N_-[2,2-bis(6-Bromo-1_H_-indol-3-yl)ethyl]thiophene-2-carboxamide (3g)

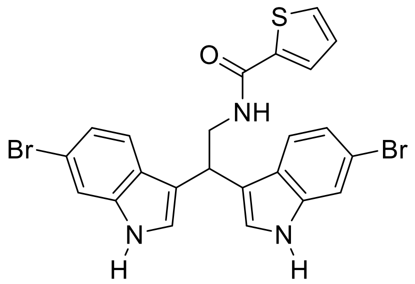

Following the general procedure **C**, compound **3g** was isolated as a white solid (*n*-heptane/EtOAc 100:0 to 25:75) in 45% yield (48 mg). ^{1}H NMR (400 MHz, $(CD_3)_2CO$) δ 10.21 (br s, 1H), 7.91 – 7.81 (m, 1H), 7.61 (dd, J = 5.0, 1.1 Hz, 1H), 7.58 (dd, J = 1.8, 0.5 Hz, 2H), 7.57 – -7.53 (m, 2H),7.51 (dd, J = 3.8, 1.1 Hz, 1H), 7.30 – 7.26 (m, 2H), 7.07 (dd, J = 8.5, 1.8 Hz, 2H), 7.03 (dd, J = 5.0, 3.7 Hz, 1H), 4.92(app t, J = 7.3Hz, 1H), 4.12 (dd, J = 7.4, 6.0 Hz, 2H). ^{13}C NMR (101 MHz, $(CD_3)_2CO$) δ 162.5, 141.3, 138.8, 130.8, 128.3, 127.1, 124.5, 124.3, 122.4, 121.7, 117.8, 115.3, 115.0, 45.2, 35.2. HRMS (TOF-ESI$^+$): *m*/*z* calcd for $C_{15}H_{12}BrN_2OS$ [M - 6-bromoindole]$^+$ 346.9854; found 346.9854. LC-MS: [M - 6-bromoindole + 2]$^+$ *m*/*z* 349 (t_r = 5.47 min), >99%.

N-[2,2-bis(6-Bromo-1H-indol-3-yl)ethyl]-4-methylbenzenesulfonamide (4a)

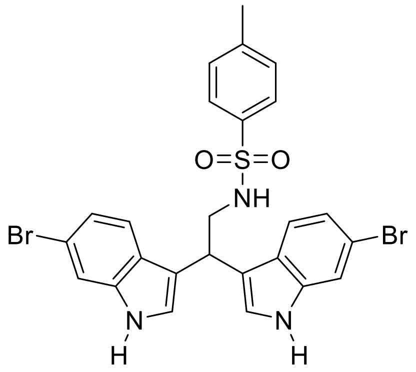

Following the general procedure **D**, compound **4a** was isolated as a white solid (DCM/MeOH 100:0 to 99:1) in 92% yield (108 mg). ^{1}H NMR (400 MHz, CD_3OD) δ 7.54 (d, J = 8.3 Hz, 2H), 7.44 (d, J = 1.7 Hz, 2H), 7.22-7.12 (m, 4H), 6.99-6.94 (m, 4H), 4.53 (app t, J = 7.3 Hz, 1H), 3.56 (d, J = 7.2 Hz, 2H), 2.35 (s, 3H). ^{13}C NMR (101 MHz, CD_3OD) δ 144.4, 139.1, 130.4, 127.8, 127.0, 124.5, 122.6, 121.3, 117.1, 115.7, 115.1, 48.4, 35.7, 21.5. HRMS (TOF-ESI$^+$): *m/z* calcd for $C_{25}H_{23}Br_2N_3O_2$ $[M+H]^+$ 585.9799; found 585.9803.LC-MS: $[M - 6\text{-bromoindole} + 2]^+$ *m/z* 393 (t_r = 6.02 min), >95%.

N-[2,2-bis(1-Methyl-1H-indol-3-yl)ethyl]-4-methylbenzenesulfonamide (4b)

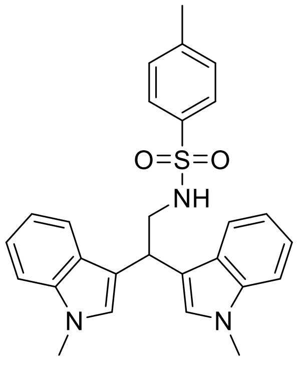

Following the general procedure **D**, compound **4b** was isolated by column chromatography as a white solid (petroleum ether/EtOAc 90:10 to 70:30) in 89% yield (62 mg). ^{1}H NMR (400 MHz, $CDCl_3$) δ 7.63 – 7.58 (m, 2H), 7.40 (d, J = 8.0 Hz, 2H), 7.29 (d, J = 8.2 Hz, 2H), 7.24 – 7.19 (m, 2H), 7.02 (ddd, J = 8.0, 7.0, 1.0 Hz, 1H), 6.82 (s, 2H), 4.64 (t, J = 6.9 Hz, 1H), 4.48 (br s, 1H), 3.70 (s, 6H), 3.69 – 3.64 (m, 2H), 2.43 (s, 3H). ^{13}C NMR (101 MHz, $CDCl_3$) δ 143.4, 137.4, 136.7, 129.8, 127.4, 127.3, 126.9, 121.9, 119.4, 119.1, 114.3, 109.5, 47.1, 34.3, 32.9, 21.7. Anal. calcd. for $C_{27}H_{27}N_3O_2S$ (457.59) C 70.87, H 5.95. N 9.18; found C 71.02, H 6.02. N 9.26.

***N*-[2,2-bis(6-Bromo-1*H*-indol-3-yl)ethyl]thiophene-2-sulfonamide (4c)**

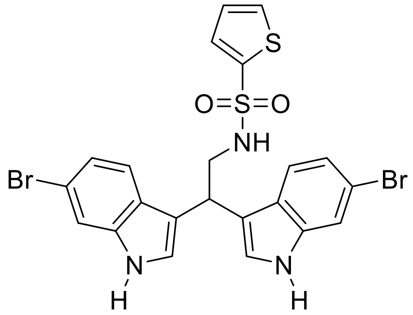

Following the general procedure **D**, compound **4c** was isolated as a white solid (DCM/MeOH 100:0 to 99:1) in 83% yield (73 mg). ^{1}H NMR (400 MHz, CD_3OD) δ 7.67 (dd, J = 5.0, 1.3 Hz, 1H), 7.48 (dd, J = 3.7, 1.3 Hz, 1H), 7.47 (dd, J = 1.8, 0.5 Hz, 2H), 7.27 (dd, J = 8.5, 0.6 Hz, 2H), 7.04 (dd, J = 5.0, 3.7 Hz, 1H), 7.02 – 6.98 (m, 4H), 4.62 (app t, J = 7.3, 1H), 3.62 (d, J = 7.3 Hz, 2H). ^{13}C NMR (101 MHz, CD_3OD) δ 143.0, 139.1, 132.8, 132.7, 128.4, 127.1, 124.5, 122.7, 121.3, 117.2, 115.8, 115.2, 49.0 (under the solvent peak), 35.9. HRMS (TOF-ESI^{+}): *m*/*z* calcd for $C_{22}H_{18}BrN_3O_2S_2$ [M + H]$^{+}$ 577.9207; found 577.9211. LC-MS: [M - 6-bromoindole + 2]$^{+}$ *m*/*z* 385 (t_r = 5.72 min), >95%.

5. References

1. Strickley RG. Solubilizing excipients in oral and injectable formulations. Pharm Res. 2004;21(2):201. 10.1023/b:pham.0000016235.32639.23
2. Li P, Zhao L. Developing early formulations: Practice and perspective. Int. J. Pharm. 2007;341:1. https://doi.org/10.1016/j.ijpharm.2007.05.049
3. Williams HD, Trevaskis NL, Charman SA, Shanker RM, Charman WN, Pouton CW, et al. Strategies to address low drug solubility in discovery and development. Pharmacol. Rev. 2013;65(1):315. https://doi.org/10.1124/pr.112.005660
4. Mantenuto S, Lucarini S, De Santi M, Piersanti G, Brandi G, Favi G et al. One-pot synthesis of biheterocycles based on indole and azole scaffolds using tryptamines and 1,2-diaza-1,3-dienes as building blocks. Eur. J. Org. Chem. 2016;2016(19):3193. https://doi.org/10.1002/ejoc.201600210
5. Mari M, Tassoni A, Lucarini S, Fanelli M, Piersanti G, Spadoni G. Brønsted acid catalyzed bisindolization of α-amido acetals: Synthesis and anticancer activity of bis(indolyl)ethanamino derivatives. Eur. J. Org. Chem. 2014:2014(18):3822. https://doi.org/10.1002/ejoc.201402055
6. Campana R, Favi G, Baffone W, Lucarini S. Marine alkaloid 2,2-bis(6-bromo-3-indolyl) ethylamine and its synthetic derivatives inhibit microbial biofilms formation and disaggregate developed biofilms. Microorganisms.2019;7(2):28. https://doi.org/10.3390/microorganisms7020028
